# An Effective Measured Data Preprocessing Method in Electrical Impedance Tomography

**DOI:** 10.1155/2014/208765

**Published:** 2014-07-22

**Authors:** Chenglong Yu, Shihong Yue, Jianpei Wang, Huaxiang Wang

**Affiliations:** School of Electrical Engineering and Automation, Tianjin University, Tianjin 300072, China

## Abstract

As an advanced process detection technology, electrical impedance tomography (EIT) has widely been paid attention to and studied in the industrial fields. But the EIT techniques are greatly limited to the low spatial resolutions. This problem may result from the incorrect preprocessing of measuring data and lack of general criterion to evaluate different preprocessing processes. In this paper, an EIT data preprocessing method is proposed by all rooting measured data and evaluated by two constructed indexes based on all rooted EIT measured data. By finding the optimums of the two indexes, the proposed method can be applied to improve the EIT imaging spatial resolutions. In terms of a theoretical model, the optimal rooting times of the two indexes range in [0.23, 0.33] and in [0.22, 0.35], respectively. Moreover, these factors that affect the correctness of the proposed method are generally analyzed. The measuring data preprocessing is necessary and helpful for any imaging process. Thus, the proposed method can be generally and widely used in any imaging process. Experimental results validate the two proposed indexes.

## 1. Introduction

Electrical impedance tomography (EIT) technique [1] is one nondestructive visualization measurement technology. Due to fast-response, noninvasive, low cost in obtaining 2D/3D distribution parameter information, EIT has been widely used in several principal areas such as medical imaging, industrial process imaging, and geophysical surveying [2]. But the EIT techniques are greatly limited to the low spatial resolutions [3–5] that greatly result from the following three problems.

(*1) Low Relative Resolution of Measurable Data.* When these measuring data are of low relative resolution, that is, too small size difference of measuring data compared with their own sizes, the reconstructed EIT image is of low spatial resolution. In fact, most of the existing EIT imaging methods depend on an optimization process associated with a good relative solution of measurable data, while this process is an ill-conditioned, highly nonlinear, and uncertain problem [6]. If there are not good relative solutions of measurable data, the reconstructed images are of low resolution. Thus, an optimal measuring data preprocessing is necessary since the ill-conditioned equation is tightly associated with the measuring data.

(*2) Low Signal-to-Noise Ratio.* The EIT measuring data are of low signal-to-noise ratio (SNR). Besides machine noise, owing to the use of weak current excitation, the measuring data in EIT must be a weak signal, and any small measuring errors or noise may lead to large spatial resolution of the investigated objects in an EIT image, or roughly speaking, any change of any objects in the investigated field could affect all measured data of all other objects in the investigated field. This is called “soft-field” effect which is a much undesired case in practice [7, 8]. Thus, “soft-field” effect is mainly responsible for the low SNR in the EIT imaging process [9, 10]. The existing ET imaging processes are unstable and often unacceptable in most noisy conditions [11, 12].

(*3) Imaging Algorithm.* A challenging problem in the EIT imaging process is how to quantitatively choose the proper algorithm to obtain the highest spatial resolution of EIT images. As a result, these reconstructed images usually do not have interpretability or understandability [13]. Some researchers attempt to solve this problem using simulation, visualization, and new hardware system for gaining comparable statistics [14, 15], but these methods are of little applicability in practice.

The importance of the preprocessing processes of measuring data has been realized. In order to obtain the EIT image of high resolution, some researches attempt to adjust the size of original measuring data based on the hardware system. For example, the measuring data are amplified by a special integrated circuit. However, these methods concentrate on a/some linear transformations to all data, while a linear transformation cannot change the relative size of these measuring data. When there are noisy data, a linear transformation will simultaneously amplify signal and noises. In this paper, we present a nonlinear transformation to all measuring data. In terms of rooting all measured data, an EIT data preprocessing method is proposed and evaluated by two constructed indexes based on all rooted EIT measured data. One index aims to maximize the relative size among measurable data, and the other one aims to maximize SNR of the measuring data. Dependent on their optimums of the two indexes, the optimal root of the measuring data can be obtained. One purpose of rooting all data is to find the optimal relative size of measurable data such that the EIT spatial resolution of the EIT imaging can be improved greatly. Another purpose of rooting the measuring data is to attain as high SNR as possible. The research in this paper shows that the two proposed indexes have nearly the same optimums but different motivations. The analytic optimal solutions of the two proposed indexes are obtained by a mathematical analysis. The analysis of the two indexes involves the existing study [16]. Recently, the natural clustering structures hidden in the EIT measuring data have been recovered and the fuzzy clustering algorithm termed as FC-EIT is proposed for EIT imaging process. The reconstructed images by the FC-EIT algorithm can obtain much higher spatial resolution in a wide range of parameter settings. Particularly, some experimental results from the FC-EIT algorithm are unable to be completed by other existing EIT algorithms at all. In this research, FC-EIT and other three existing EIT imaging algorithms are applied to test the correctness of the two proposed indexes.

The rest of this paper is organized as follows.  Section 2 introduces the EIT imaging principle. In Section 3, the two indexes for measuring data preprocessing are proposed and analyzed in different conditions. Experimental tests and results of the two indexes are interpreted in Section 4.  Section 5 is the conclusion.

## 2. EIT Imaging Principle

**Figure 1:**
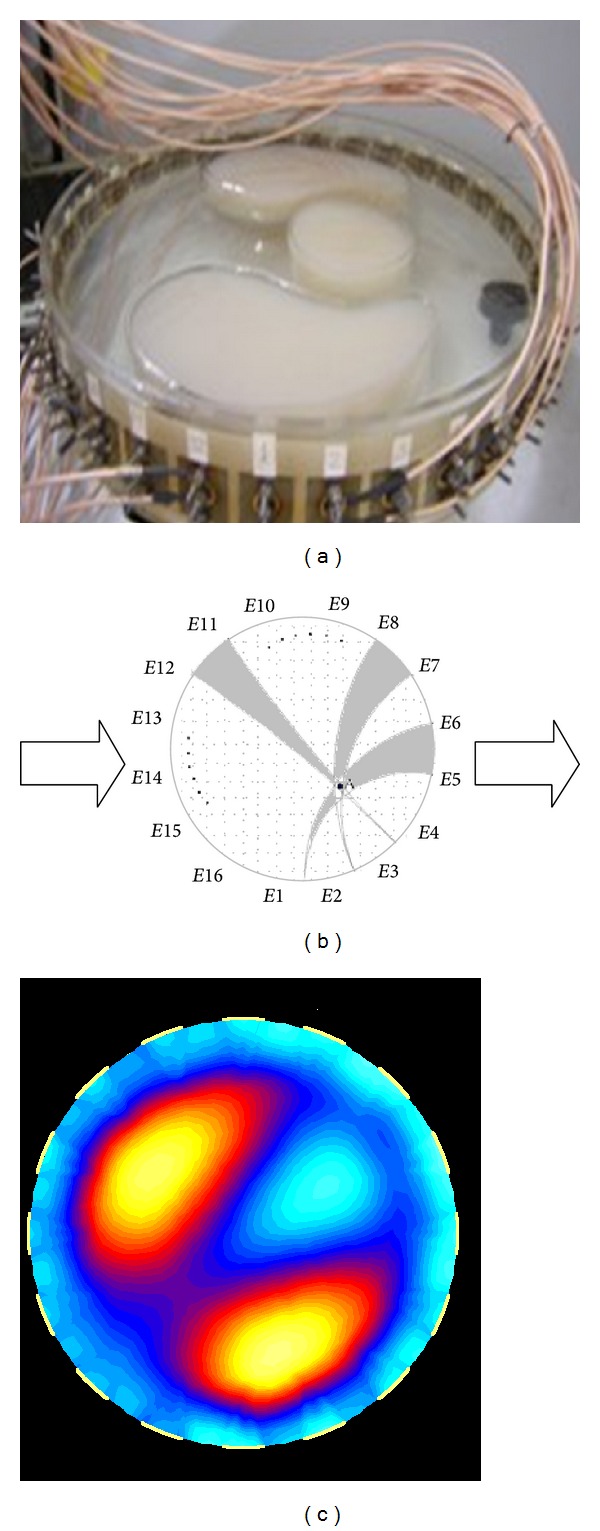
EIT imaging process. (a) Original investigated objects of three blocks of watered agars. (b) Each pixel is covered by 16 projection fields (in grey) from 16 excitations. (c) Reconstructed EIT image by the LBP algorithm based on measuring data.

As an example, an ERT system of a total of 16 electrodes evenly distributed around a 16-centimeter radius pipe is applied to illustrate the EIT imaging structure and process. Original investigated objects are three blocks of watered agars that are put in salt water, as shown in Figure 1.

Figure 1(a) shows the investigated objects and the distribution of 16 electrodes in an ERT system. The adjacent exciting strategy is used for data collection, that is, among a total of 16 pairs of electrodes when the exciting current is added to one electrode pair (*i*, *i* + 1), 15 voltage values of other electrodes are measured, *i* = 1, 2,…, 16. After excitation electrode pair is switched 16 times, 16 groups of measurements are obtained among which two measurements including excitation electrode pairs need to be discarded due to the large errors. Consequently, 13 groups of measurements in each excitation are used for a frame of EIT image reconstruction. To reconstruct a frame of ERT image, the cross-section is discretized by rectangular or triangular units related to pixels in the ERT image (see Figure 1(b)). The cross-section boundary and any pair of equipotential lines connected to two adjacent electrodes construct a projection field. Each extraction electrode corresponds to 16 projection fields (in grey) over the entire cross-section, and thus any pixel in the cross-section must be covered by 16 projection fields, respectively, from 16 different extraction electrodes. In terms of these measuring data, the investigated objects are visually reconstructed (see Figure 1(c)).

The EIT imaging process obeys the general Maxwell equation [5] whose simplified physical model for EIT can be written as

(1) $$\begin{array}{c} U=f\left(\sigma ;I\right)=R\left(\sigma \right)I, \end{array}$$

where *U* is the measured voltage vector on the electrodes surrounding the periphery of a subject, *I* is the injected current vector, *σ* is the conductivity distribution in a cross-section of the subject, *f*(*σ*; *I*) is the nonlinear model mapping *σ* and *I* to *U*, and *R*(*σ*) is the model mapping *σ* to resistance. This model depends nonlinearly on the conductivity *σ* and linearly on the current *I*. The aim of image reconstruction for EIT is to obtain the conductivity distribution *σ* using the boundary voltage vector *U* and the injected current vector *I*. The mathematical expression of the tomographic problem is given by the following equations:

(2) $$\begin{array}{c} {\left[\Delta U\right]}_{K\times 1}={\left[U\right]}_{K\times N}\cdot {\left[\Delta G\right]}_{N\times 1}\;\text{or}\;\;U=SG, \end{array}$$

where *S* is a Jacobian matrix, that is, the sensitivity distribution, and  *K* and *N* are the numbers of total measurements and pixels, respectively. The goal is to determine the unknown image *X* when the experimental projections *U* are available. In the discrete form, the aim of image reconstruction for the EIT field is to find the unknown pixel vector from the known *U* by using (2); that is,

(3) $$\begin{array}{c} G=S^{-1}U. \end{array}$$

However, the direct analytical solution for (3) does not exist since the inverse problem is both nonlinear and ill-posed, and little noise in the measured data could cause large errors in the estimated conductivity. Many algorithms have been proposed to indirectly solve the above ill-posed problem as explained below.

(*1) LBP Algorithm.* The most used EIT image reconstruction algorithm is the linear back projection (LBP) [6]. In the LBP algorithm, the conductivity distributions are assumed to comprise a number of discrete regions within the measurement space such that the conductivity within each region is constant. According to (3), *A* ^−1^ *B* is approximated as

(4) $$\begin{array}{c} G=\frac{S^{T}U}{S^{T}U_{\lambda }},\;\text{s.t.}\;\;\;U_{\lambda }=\left[1,1,\ldots ,1\right]. \end{array}$$

Equation (4) shows that the grey value of any pixel is calculated by using a weighted form in the LBP algorithm.

(*2) Landweber Algorithm.* The Landweber algorithm (LW) [14] was originally designed for solving the classical ill-posed problem using the strategy similar to the gradient descending algorithm in the optimization process by the following equation:

(5) $$\begin{array}{c} G_{t+1}=G_{t}-\alpha S^{T}\left(SG_{t}-\lambda \right), \end{array}$$

where the constant *α* is known as the gain factor and is used to control the convergence rate. As the iterative process described by (5) proceeds, the norm of the capacitance residual will be minimized. Since the norm may tend to be a certain value larger than zero, the original algorithm often is modified as

(6) $$\begin{array}{c} G_{t+1}=P\left[G_{t}-\alpha S^{T}\left(SG_{t}-\lambda \right)\right]. \end{array}$$

The value of *P* has been adopted by inclusion of a nonlinear function to constrain the estimated image so that *G*_*t*+1_ ∈ [0,1]; that is, when a normalized gray level is less than zero, it is constrained to be zero, and when it is larger than “1”, it is constrained to be “1”.

(*3) Tikhonov Regularization.* The Tikhonov regularization (TR) [15] is one efficient method and is presented as a minimization function shown as follows:

(7) $$\begin{array}{c} J\left(g\right)=\frac{1}{2}{||U-S\sigma ||}^{2}+\mu R\left(\sigma \right), \end{array}$$

where *R*(*g*) is the regularization function and *μ* is the regularization parameter. The function is often expressed in *L* ^2^ form as

(8) $$\begin{array}{c} R\left(g\right)={||L\left(\sigma -\overset{-}{\sigma }\right)||}^{2}, \end{array}$$

where *L* is an appropriate regularization matrix and $\overset{-}{\sigma }$ is a prior estimate of the permittivity or the conductivity distribution.

(*4) FC-EIT Algorithm.* Recently, one efficient and original EIT imaging algorithm [16], termed as FC-EIT, has been proposed. FC-EIT first maps *K* measuring data of pixel *j* after *K* excitations into a *K*-dimensional vector as

(9) $$\begin{array}{c} {\left(v_{j,1}v_{j,2},\ldots ,v_{j,K}\right)}^{T},\;\forall j=1,2,\ldots ,n. \end{array}$$

All vectors associated with all pixels consist of a set *X* and then all vectors in *X* are partitioned into *c* clusters by the fuzzy *c*-means (FCM) algorithm [17]. Let *u*_*ij*_ be the membership degree of *j*th vector (pixel) to *i*th cluster, let *v*_*i*_ be the cluster prototype of *i*th cluster, and let *G*(*v*_*i*_) be the gray value of *i*th cluster prototype, *i* = 1,2,…, *c*; *j* = 1, 2, …, *n*. So the gray value of *j*th pixel *g*(*j*) is determined by the weighted average form:

(10) $$\begin{array}{c} g\left(j\right)=\overset{c}{\underset{i=1}{\sum }}u_{ij}G\left(v_{i}\right),\;j=1,2,\ldots ,n. \end{array}$$

All pixels are endowed with different gray values based on (10). These determined gray values thus can reconstruct a frame of EIT image to show various conductivities and to recover the distributions of investigated objects in the cross-section.

LBP is a typical noniterative algorithm and has the least excusive time, and the others are iterative algorithms and need more runtime. These EIT imaging algorithms are the most used ones in practice. In this paper, the above algorithms are applied for EIT imaging reconstruction to examine the spatial resolutions before and after performing measuring data preprocessing.

## 3. Optimal Data Preprocessing Method

In this section, we define a relative size (RS) index and a signal-to-noise ratio (SNR) index of the measureable data, respectively. The optimums of the two indexes are applied to realize the optimal measuring data preprocessing and improve the EIT spatial resolution. The characteristics of the two indexes are illustrated below.

### 3.1. Two Quantitative Indexes for Evaluating Data Preprocessing Process

The relative sizes of *K* measuring data of any pixel after *K* excitations can lead to great differences for the EIT spatial resolution. Due to the weak current excitation in EIT, most of the measuring data have very small size. Thus, amplifying the original data to a proper size is a necessary step for the EIT imaging process [2, 12]. The larger measureable data relative size, the higher EIT images spatial resolution [12, 16]. The first proposed index aims to maximize the relative size by using a nonlinear transformation of all measuring data, because a linear amplification must keep the relative sizes being a constant. Assuming that an investigated field is partitioned into *n* pixels, a nonlinear transformation is performed by finding *z* root to all measuring data as in the following form:

(11) $$\begin{array}{c} y_{k,j}=\sqrt[{z}]{v_{k,j}},\;k=1,\ldots ,K;\;\;j=1,2,\ldots ,n, \end{array}$$

where *v*_*k*,*j*_ is the measured data of the *j*th pixel in the *k*th excitation, *k* = 1, 2, …, *K*. The proposed index, termed as the relative size (RS) index, is defined as

(12) $$\begin{array}{c} \xi =\overset{n}{\underset{j=1\;}{\sum }}\overset{K}{\underset{s,t=1}{\sum }}\frac{\left(\max\left\{y_{j,s},y_{j,t}\right\}-\min\left\{y_{j,s},y_{j,t}\right\}\right)}{n\left(K-1\right)}, \end{array}$$

where *s* and *t* are two consecutive integers, and *v*_*j*,*s*_ and *v*_*j*,*t*_ are two successive measuring data from the two successive excitations. The value of *ξ* totally reflects the relative size of measuring data of all pixels. To maximize the relative size, the optimum of (12) is solved as

(13) $$\begin{array}{c} z^{*}=\arg\;\text{ma}{\text{x}}_{z}\xi . \end{array}$$

Independent of any measuring data and any EIT imaging algorithms, (13) must have a maximum at least for any set of pixels associated with these investigated objects due to the following three reasons.

- For  *ξ*≧0, the inequality holds from (12).
- For  *ξ* = 0 when *z* → *∞*, *y*_*ij*_ → 1, the equation holds since zero exponents of any number are 1 and thus are equivalent to each other.
- For  *ξ* ≈ 0 when *z* → 1, all measuring data keep their original values and the mutual differences of these data are very small.

Equation (13) shows that the optimal measuring data can be solved by the optimum of the RS index of (13). Thus, the optimal determination of the rooting times of the measuring data can enhance their relative sizes.

**Figure 2:**
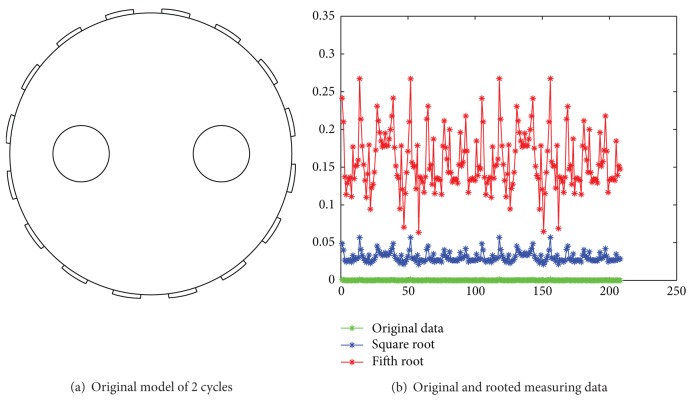
Extraction of roots for the measured data.

Figure 2(a) shows an original image that is simulated in Comsol simulation environment [18]. The reconstructed objects are two circles with conductivity 200 and the corresponding background with conductivity 100.  Figure 2(b) shows the simulated measurable data associated with the original image. It is difficult to identify these measuring data in terms of their original relative sizes. As compared, the second and the fifth roots of these measurable data are shown in the same coordinate. Compared with the original measuring data, their relative sizes become larger. In fact, their values of *ξ* are 12.12 and 19.30, respectively, while the value of *ξ* of the original measuring data is 2.73. This demonstrates that extracting the root of measurable data can increase their relative sizes.

On the other hand, for *K* measuring data of any pixel, there are seriously noisy data and “soft-field” effects. Usually, the two measuring data that are closest and farthest to a pair of exciting electrodes are of low SNR. The closest one is due to the strong proximity effect between exciting and measuring electrodes, and the farthest one is prone to be affected by these polarization effects and “soft-field” effects in the field. Consequently, these measuring data that are nearly located in the middle between the closest and farthest electrodes are the most effective among *K* measuring data. The second index aims to minimize the effect of the two measuring data by finding their optimal root by (11). So the second index, termed as* the* SNR* index*, is defined as

(14) η=2nK×∑j=1n∑k=1Kvj,kmax⁡⁡{vj,1,vj,2,…,vj,k}+min⁡{vj,1,vj,2,…,vj,k},

where max⁡{*v*_*j*,1_, *v*_*j*,2_,…, *v*_*j*,*k*_} and min⁡{*v*_*j*,1_, *v*_*j*,2_,…, *v*_*j*,*k*_} are the farthest and closest measuring data in *K* measuring data, respectively. The coefficients of 2/*nK* make the values of *η* equal to 1 when all values of *v*_*j*,*k*_ are equivalent to each other. It is clear that the larger the values of *η* are, the higher the SNR is. Thus, values of *η* can show the SNR level of these measuring data. To obtain the highest SNR, (14) is solved by

(15) $$\begin{array}{c} z^{*}=\arg\;\text{ma}{\text{x}}_{z}\eta . \end{array}$$

The optimum of (15) shows the SNR level of the measuring data after finding the optimal root. Equation (15) must have a maximum at least for any set of pixels associated with these investigated objects due to the following three reasons.

- For  *η*≧1, the inequality holds from (14).
- For  *η* ≈ 1 when *z* → 1, all measuring data keep their original values and the mutual differences of these data are very small.
- ) For  *n* = 1 when *z* → *∞*, *y*_*ij*_ → 0, the equation holds since the infinite roots of any numbers are 1 and thus are equivalent to each other.

**Figure 3:**
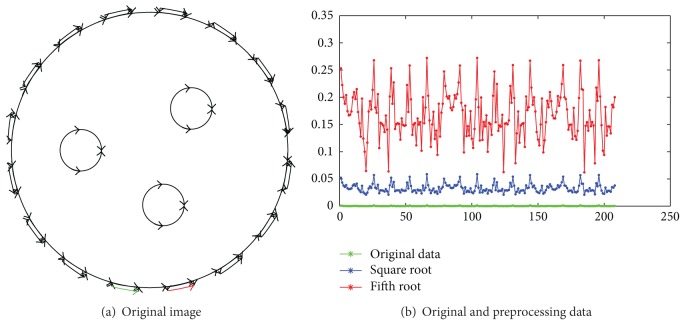
Original image and effects of rooting measuring data.

Figure 3 shows a simulation in Comsol simulation environment [18]; the original image consists of three circles with the same conductivity as well as background. The simulated measuring data shown in their optimal 3rd root. Compared with the original measuring data, the data after finding their roots have much larger values of *η*. This demonstrates that the rooting operation is helpful in improving the SNR level.

### 3.2. Optimal Solution of the Two Proposed Indexes

**Figure 4:**
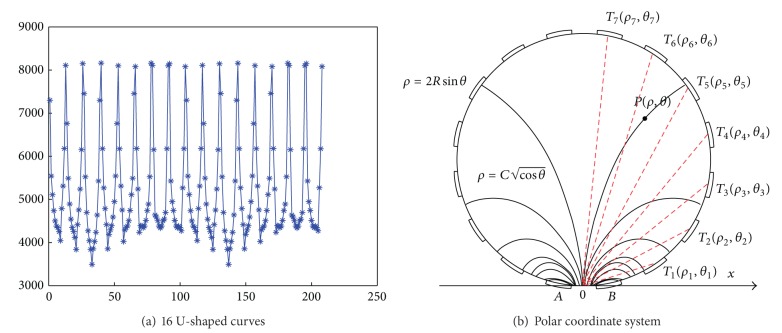
Measuring data with U-shaped curve and polar coordinate system.

The cross-section in an EIT system is called a full field when the investigated objects are contained and otherwise is called an empty field. The optimums of (13) and (15) are solved in a full field. However, it is impossible to obtain an analytic optimum for an arbitrary full field owing to extremely complex distributions of investigated objects. Notice that the relative sizes of measuring data in a full field are nearly proportional to those in the empty field [12, 13]. Consequently, the optimums of (13) and (15) are solved in an empty field. Without loss of generalization, a system of 16 electrodes is taken in a circular investigated field as an example. For each excitation, 13 measuring data consist of a U-shaped curve and all measuring data from 16 excitations are summed up to 208 for the same pixel, as shown in Figure 4(a). Assume that *T*_1_, *T*_2_, *T*_3_, *T*_4_, *T*_5_, *T*_6_, and *T*_7_ are 7 electrodes on a circular empty field, as shown in Figure 4(b).

The circle equation with radius *R* in a polar coordinate system can be formulated as

(16) $$\begin{array}{c} \rho =2R\sin\theta , \end{array}$$

where *ρ* is polar radius and *θ* is polar angle. Each pair of exciting electrodes *A* and *B* is regarded as an electric dipole since they have enough small distance compared with the radius of the circled field. The polar coordinate equation on the equipotential line in arbitrary *P* point in the investigated field is shown as

(17) $$\begin{array}{c} \rho =C\sqrt{\cos\theta }, \end{array}$$

where *C* is the potential value of the equipotential line that goes through *P* point. According to the basic mathematical theorem [19], the same arc has equivalent angle in a circular segment, drawing out the following relation:

(18) $$\begin{array}{c} ∠T_{1}OT_{2}=∠T_{2}OT_{3}=\cdots =∠T_{6}OT_{7}. \end{array}$$

Let the polar coordinates *T*_1_, *T*_2_,…, *T*_7_ be (*ρ*_1_, *θ*_1_), (*ρ*_2_, *θ*_2_),…, (*ρ*_7_, *θ*_7_) after the polar point is taken as the center of the exciting electrodes *A* and *B*, respectively. To begin with *x*-axis at the *k*th excitation, these polar angles *T*_1_, *T*_2_,…, *T*_7_ are represented as

(19) $$\begin{array}{c} \theta_{i}={\left(\frac{2\pi }{15}i\right)}^{0}\approx {\left(24i\right)}^{0},\;i=1,2,\ldots ,7. \end{array}$$

After combining (16) and (17), the potential values of *T*_1_ ~ *T*_7_ are solved as

(20) $$\begin{array}{c} C=\frac{2R\sin\theta }{\sqrt{\cos\theta }}⟹C_{i}=\frac{2R\sin\theta_{i}}{\sqrt{\cos\theta_{i}}}=Q_{i}R,\;i=1,2,\ldots ,7, \end{array}$$

where $Q_{i}=2\sin\theta_{i}/\sqrt{\cos\theta_{i}}$, *i* = 1,2,…, 7, are 7 invariant constants when the number of electrodes *K* is fixed. The potential relative sizes of these measuring data among the seven electrodes *T*_1_,…, *T*_7_ are

(21) vj,k=uj,k+1−uj,k=(Qj,i+1−Qj,i)R, j=1,2,…,n, k=1,2,…,7.

Generally, owing to *v*_*j*,1_ > *v*_*j*,2_ > ⋯>*v*_*j*,7_ and $\sqrt[{z}]{K_{j,2}-K_{j,1}}>\sqrt[{z}]{K_{j,3}-K_{j,2}}>\cdots >\sqrt[{z}]{K_{j,K+1}-K_{j,K}}$, therefore (13) and (15) can be rewritten as

(22) $$\begin{array}{c} \xi =\overset{n}{\underset{j=1\;}{\sum }}\overset{k}{\underset{s,t=1}{\sum }}\frac{\left(\sqrt[{z}]{Q_{j,s+1}-Q_{j,s}}-\sqrt[{z}]{Q_{j,t+1}-Q_{j,t}}\right)\sqrt[{z}]{R}}{n\left(K-1\right)},\\ \eta =\frac{2}{nK}\overset{n}{\underset{j=1}{\sum }}\frac{\sum_{k=1}^{K}\sqrt[{z}]{Q_{j,k+1}-Q_{j,k}}}{\sqrt[{z}]{K_{j,3}-K_{j,2}}+\sqrt[{z}]{K_{j,K+1}-K_{j,K}}}. \end{array}$$

When the number of electrodes is fixed, the optimums of (13) and (15) are two constants and can be solved easily. According to the finite element method [20], when *K* = 16 for ERT and *K* = 12 for ECT, the optimums of (1/*z*) in (13) are 0.26 and 0.34, and the optimums in (15) are 0.23 and 0.33, respectively.

**Figure 5:**
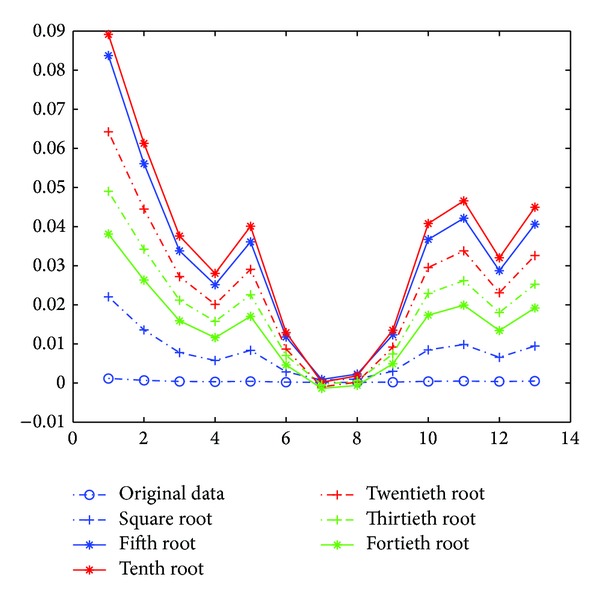
Variance of U-shaped curves after rooting the measuring data.

According to the two original images in Figures 2 and 3, Figure 5 shows the optimal solutions of (13) and (15). Figure 5 shows that the relative sizes of the measuring data gradually increase from 0.1 to 0.26 and decrease from 0.26 to 0.1, where all origins of U-shaped curves after rooting these measuring data are located in the same point.

Both (13) and (15) can provide the optimal measuring data preprocessing according to the same rooting operation in (11) but are based on different motivations. In the experimental part in this paper, our research shows their interrelations.

### 3.3. The Correctness of the Two Proposed Indexes

The optimums of the two proposed indexes are solved under this assumption that variances of the measuring data are nearly directly proportional to each other in the empty and full fields. However, a real investigated field can be affected by various and complex applicable conditions, and thus it is necessary to consider the correctness of the two indexes in these conditions. Generally, the investigated objects in a field consist of materials with different attributes, such as conductivity, permittivity, and permeability. The materials of the same attribute must have the similar distribution of the measuring data, while the ones of different attributes have different distributions. The same distributions of measured data correspond to the same cluster, while different distributions correspond to different clusters. Consequently, the task of ET imaging aims to find all clusters of measuring data [12, 16]. Most of the actual investigated objects consist of clusters with various characteristics such as sizes, densities, and positions. So in this paper the three characteristics are generally defined to evaluate the effect of the two proposed indexes as follows.

The quantity to show position characteristic is computed as

(23) $$\begin{array}{c} \text{position}\left(c\right)=\overset{c-1}{\underset{i=1}{\sum }}\overset{c}{\underset{j=i+1}{\sum }}\frac{\left\{\max\left(P_{i},P_{j}\right)/\min\left(P_{i},P_{j}\right)\right\}}{C_{c}^{2}}, \end{array}$$

where *P*_*k*_ represents the minimal distance to the *k*th cluster from other clusters and is computed by all pairwise distances from the *k*th cluster to the closest cluster, for *k* = 1,2,…, *c*. Clearly, position(*c*) > 1 since the value of max⁡(*P*_*i*_, *P*_*j*_)/min⁡(*P*_*i*_, *P*_*j*_) must be larger than 1. Values of position(*c*) are smaller and distributions among clusters are more consistent and symmetric. Thus, values of position(*c*) can efficiently show the characteristics of relative position among all clusters.

The quantity to represent size characteristic is computed as

(24) $$\begin{array}{c} \text{size}\left(c\right)=\overset{c-1}{\underset{i=1}{\sum }}\overset{c}{\underset{j=i+1}{\sum }}\frac{\left\{\max\left(S_{i},S_{j}\right)/\min\left(S_{i},S_{j}\right)\right\}}{C_{c}^{2}}, \end{array}$$

where *S*_*k*_ refers to the size of *k*th cluster and is computed by the average of all pairwise distances between the two data vectors in *k*th cluster, for *k* = 1,2,…, *c*. Clearly, size(*c*) > 1 since the value of max⁡(*S*_*i*_, *S*_*j*_)/min⁡(*S*_*i*_, *S*_*j*_) is larger than 1. Values of size(*c*) are smaller and sizes among clusters are more consistent. Thus, values of size(*c*) can efficiently show the characteristics of relative size among all clusters.

The quantity to show density characteristic is computed as

(25) $$\begin{array}{c} \text{density}\left(c\right)=\overset{c-1}{\underset{i=1}{\sum }}\overset{c}{\underset{j=i+1}{\sum }}\frac{\left\{\max\left(D_{i},D_{j}\right)/\min\left(D_{i},D_{j}\right)\right\}}{C_{c}^{2}}, \end{array}$$

where *D*_*k*_ represents the density of *k*th cluster and is computed by a division between the number of data vectors and *D*_*k*_ in the *k*th cluster. Clearly, density(*c*) > 1 for the value of max⁡(*D*_*i*_, *D*_*j*_)/min⁡(*D*_*i*_, *D*_*j*_) is larger than 1. Smaller values of density(*c*) show that all clusters have nearly the same number of data vectors. Thus, values of density(*c*) can efficiently show the characteristics of relative density among all clusters.

**Figure 6:**
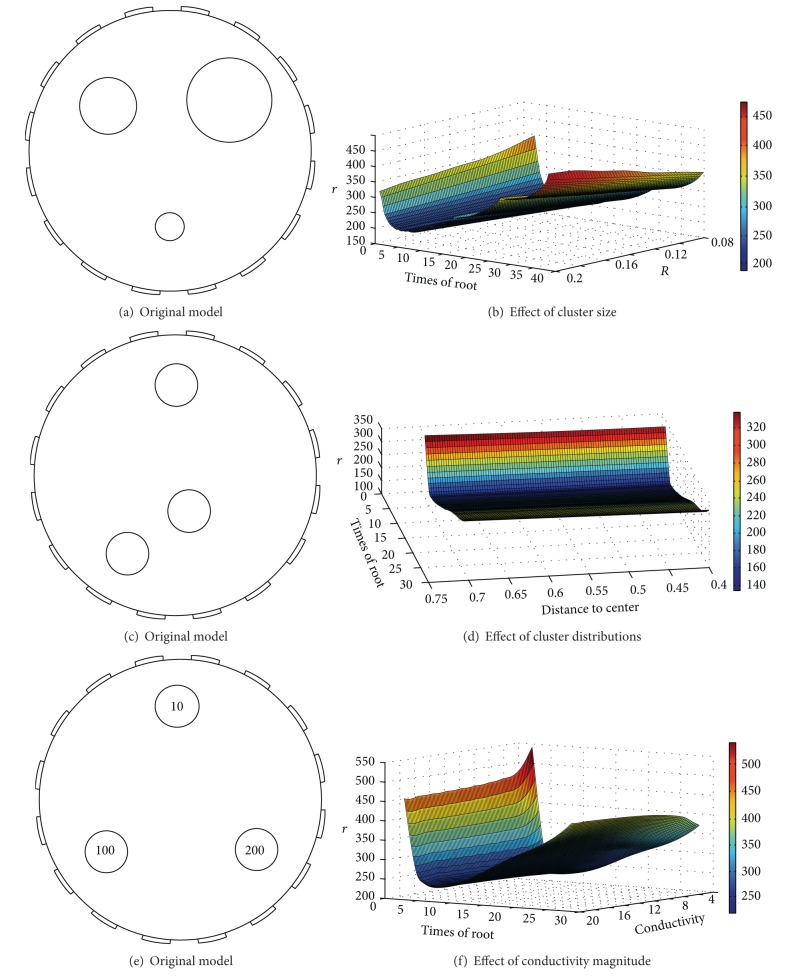
Effect of various characteristics on the optimum of the RS index. (a), (c), and (e) are six original images, and (b), (d), and (f) are the curves of the optimums by the index.

To begin with a dataset of three clusters, we reconstruct 220 three cluster-contained datasets of different characteristics by changing one quantity and changing a pair of quantities related to the above three characteristics, respectively. Figures 6(a), 6(c), and 6(e) show three representative datasets when changing one of the three quantities, respectively. As these quantities are changed, their determined optimums of the RS index are shown in Figures 6(b), 6(d), and 6(f).  Figure 6 shows that in a wide sampling range, position(*c*)∈[1, 3], size(*c*)∈[1, 4], and density(*c*)∈[1, 4] each quantity in itself has little effect on the determined optimum of the proposed index.

**Figure 7:**
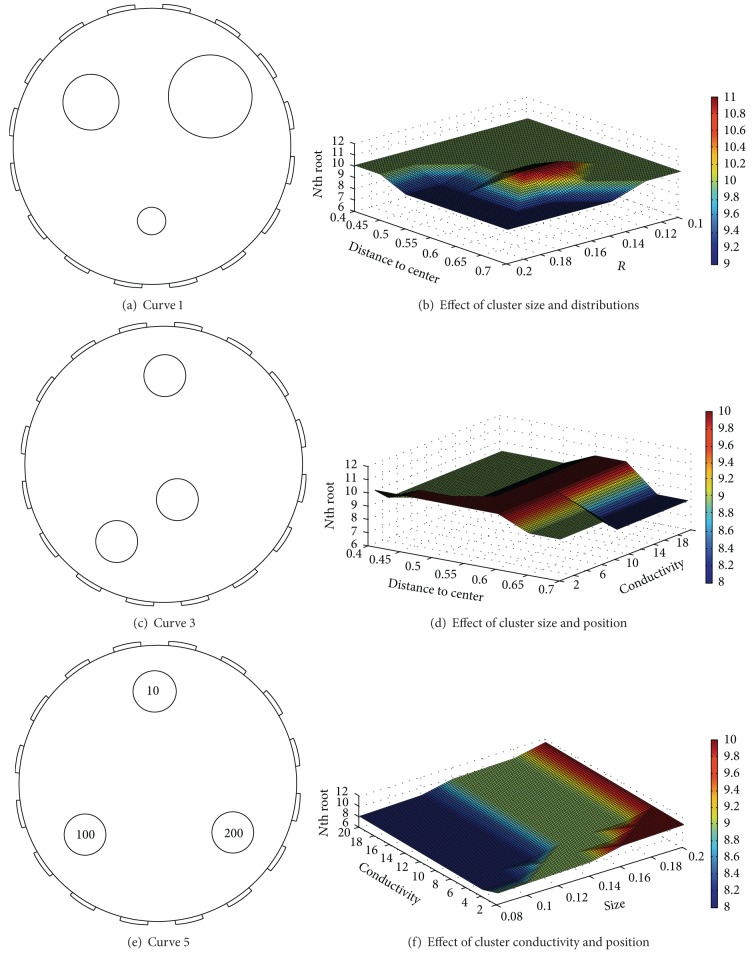
Effect of various three pairs of characteristics on the optimum of the RS index. (a), (c), and (e) are six original images, and (b), (d), and (f) are the curves of the optimums by the RS index.

Figures 7(a)–7(c) show the effect of three pairs of combined quantities for the optimums of the RS index. When position(*c*) > 3.1 and size(*c*) > 2.8, the optimum of the RS index is 0.24; when size(*c*) > 3.6 and density(*c*) > 3.8, the one is 0.26, and for position(*c*)∈[1, 3] and size(*c*)∈[1, 4] the one is around 0.3. When the above three pairs of combinations take the other values, the determined number of all clusters is 5. Thus, the combined quantities of position(*c*) and size(*c*) play a major role in the optimum of the RS index, size(*c*) and density(*c*) play a second important role, and position(*c*) and density(*c*) have no apparent effect on the final optimums. Please note that position(*c*)∈[1, 3], size(*c*)∈[1, 4], and density(*c*)∈[1, 4] are very general conditions and are encountered in most real applications.

In sum, the RS index has the following key characteristics. The optimum of the RS index can keep unchangeable in a wide range of various characteristics and thus is robust to satisfy the general needs in practice. If the number of exciting electrodes is fixed, this optimum is a constant. The optimum of the RS index assures the effectiveness and generalization of the EIT imaging process. The SNR index behaves as the RS index but they have different motivations and applicable ranges. The experimental part in this paper will present their relative sizes and interrelations.

## 4. Experiments

Two groups of experiments are applied to validate the two proposed indexes in Comsol simulation and real test, respectively. The spatial resolution of EIT sensitive field is defined as the total relative error of all pixels in the reconstructed EIT image and formulated as

(26) $$\begin{array}{c} \xi =\left(\frac{1}{K}\right)\overset{K}{\underset{j=1}{\sum }}\frac{\left(g_{j}-g_{j}^{*}\right)}{g_{j}^{*}}, \end{array}$$

where *g*_*j*_ is the reference gray value of the *j*th pixel and is known as a prior or real measuring data. *g*_j_* is the gray value of the *j*th pixel after an EIT image, *j* = 1, 2,…, *K*, and *ξ* is the average of the total error of all the *K* pixels.

The four algorithms, LBP, TR, LW, and FC-EIT, are applied to the EIT imaging process to test the correctness of the two proposed indexes.

### 4.1. Simulation in Comsol Environment

The group of experiments is implemented in Comsol simulation environment [18]. An EIT system of 16 electrodes is set up. The original images consist of two, three, four, and five circles with continuously distributed materials, respectively, as shown in the first row in Figure 14. These circles have the same conductivity and thus should be shown as the same gray degree in any EIT image, while the background has another gray value. The ratio of conductivity between the circles and background is set to 4 : 1. By the adjacent excitation way, the measuring data are produced. According to (12) and (15), we take the optimal values of the RS index and the SNR index individually as *z* = 3.5 and *z* = 3.9. In terms of these roots to all measuring data, these circles in these original images are reconstructed. Figure 14 shows these circles before and after finding the optimal root to all measuring data. Compared with the original images, the reconstructed circles based on the RS index are clear and nearly consistent after rooting all measuring data. Additionally, the trail traces in an EIT image can visually evaluate the spatial resolution of the image. It can be observed that the trail traces in these EIT images are widely distributed without data preprocessing, and even some circles are incorrectly connected to the same area. Particularly, Figure 14 shows that the Landweber algorithm can distinguish these circles better than the other three algorithms, and reconstructed images have much smaller trail traces under a wide range of parameter settings. These results further demonstrate that the Landweber algorithm outperforms the other three algorithms in the four groups of datasets and has much smaller trail traces. In fact, the average spatial resolution of these images can be raised by 17.5% by finding the optimal root of measuring data. As compared, when *z* = 3.9, the SNR index can improve spatial resolution about 12.7% according to (26). Notice that the Comsol simulation is set without noisy data, and thus the RS index may give higher improvement than the SNR index. On the other hand, the highest resolutions of the four models are 3.4, 3.8, 4.5, and 2.7, respectively. The error of the optimum is assured by the assumption that the actual data is directly proportional to the real increment of measuring data. But it should be noted that the two proposed indexes have no mathematical basis at present and this can be further improved.

**Figure 8:**
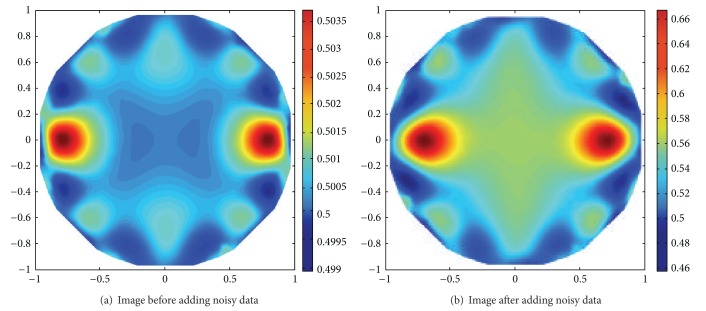
Contrast of the reconstructed images after and before adding the optimal root.

When the noisy data are added into the measured data, the spatial resolution of the reconstructed EIT must reduce. But the proposed method of rooting operation can slightly be affected and is far away from the optimums about 10%.  Figure 8 shows two reconstructed EIT images before and after 15% noisy data, where the optimal rooting values are 0.38 and 0.34 for RS and SNR, respectively, while their theoretical optimal solutions of (13) and (15) are 0.36 and 0.35, respectively.

### 4.2. Test on an ECT Field

**Figure 9:**
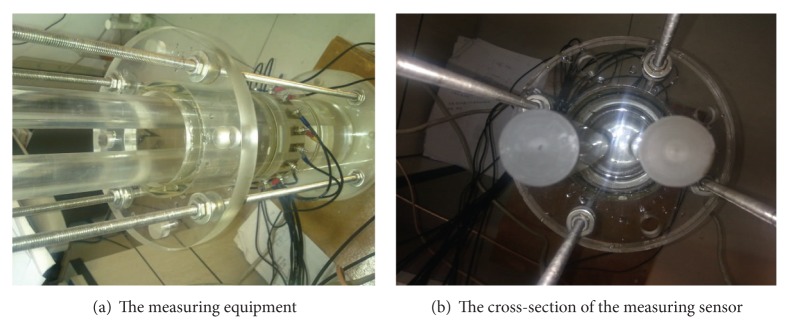
The measuring equipment and investigated objects.

In the experiment, two movable glass rods are inserted into a measuring pipe, and the background material is air, as shown in Figure 9. A 16-electrode ECT sensor is equipped on a cross-section of the pipe to excite and obtain the measuring data. The measuring process is repeated 20 times for different positions of the two rods to form data sequence sampling. There are noisy signals in this sampling process. Thus, the SNR index is applied to measuring data preprocessing to decrease the effect of the noisy signal as much as possible.

**Figure 10:**
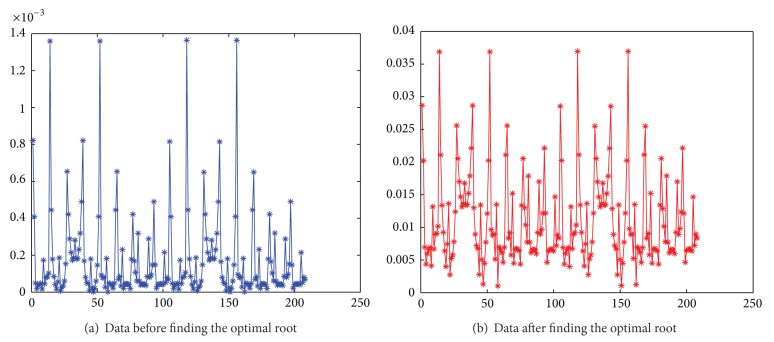
Contrast of the measured data after and before finding the optimal root.

Figure 10 shows the measuring data distribution before and after finding their optimal roots. According to the optimum of the SNR index, the measuring data before finding the optimal root are irregular and some of them deviate from the traces of U-shaped curve. Particularly, some measuring data are mixed with each other due to quite small relative size. Thus, these data may produce trail traces in the reconstructed EIT images and inevitably decrease the spatial resolution. Such partial data are caused by the machine noise in the sampling process besides the “soft-field” effect. Instead, after finding the optimal root, these measuring data become regular. Their relative sizes become larger than those before finding the optimal root.

**Figure 11:**
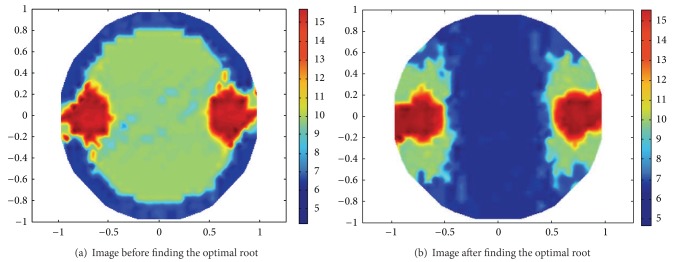
Contrast of the EIT images after and before finding the optimal root.

In terms of these measuring data before and after finding the optimal root, the LBP algorithm is applied to reconstruct the two rods, as shown in Figure 11. The two reconstructed rods after finding the optimal root are much clearer and tidier than before. According to the values of (26), in 20 times of experiments, the former averagely is 0.35 and the latter is 0.47. Consequently, the spatial resolutions of these EIT images are greatly improved based on the SNR index. In addition, the proposed data preprocessing process hardly needs to be at the expense of extra runtime since the process to find the optimal root needs much less runtime than the EIT imaging process itself.

**Figure 12:**
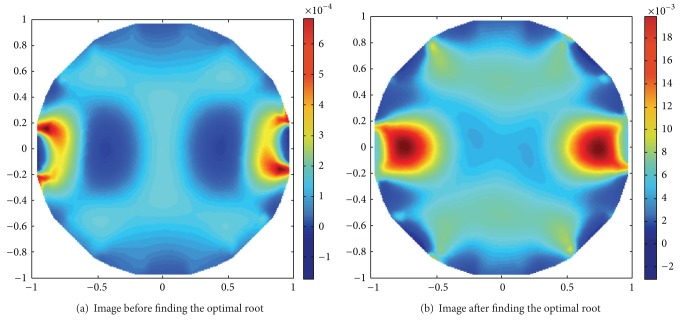
Contrast of the reconstructed images after and before finding the optimal root.

When the RS index is applied to data preprocessing and the LBP algorithm is applied to reconstruct the two rods, as shown in Figure 12, the increment of spatial resolution of the EIT image from the RS index is not as large as that from the SNR index. In fact, the values of (26) based on the RS index are averagely 2.33. Thus, the SNR index is more suitable for the noisy condition, while the RS index is more accurate when measuring data conclude little noise or are simulated data. When the rooting times in the RS index are taken from 0.2 to 0.8, the spatial resolutions of all reconstructed images can be improved with different extents. Thus, the rooting operation is a very useful data preprocessing method for the original measuring data.

**Figure 13:**
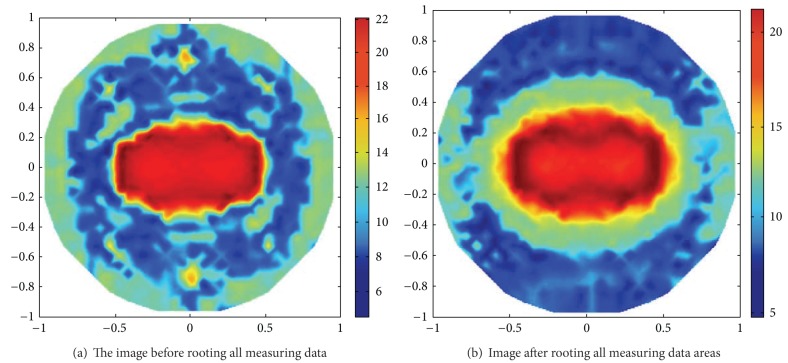
Contrast of the reconstructed images after and before finding the optimal root.

**Figure 14:**
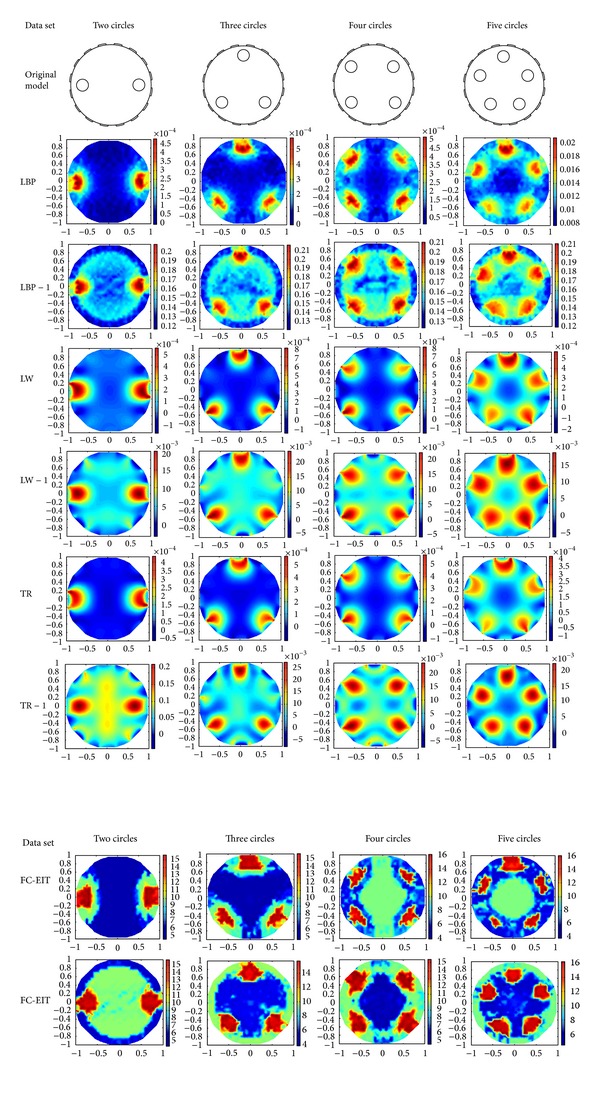
Test on the effect of finding the optimal root of measuring data. Note. “*x*” and “*x* − 1” are the EIT images before and after rooting the measuring data, respectively.

When the target objects are located in centric and boundary areas, respectively, the optimal rooting values have approximately the same results even though the spatial resolutions in the two areas are very different. But the reformulations of the spatial resolution are very limited for the EIT images. In the average meaning, the spatial resolution in the centric area reduces to 60% as much as that in the boundary area, as shown in Figure 13.

## 5. Conclusion

In this paper, a nonlinear transformation method of optimally rooting all measuring data is proposed to improve the EIT spatial resolution. The optimal rooting times are determined by two constructed indexes for measuring data preprocessing. The rooting operation has the following advantages.

(*1) Easy Operation.* The simple rooting operation of measuring data is very easy to be implemented in software and hardware systems. Moreover, various EIT techniques can follow the same way in practice.

(*2) Robustness.* The spatial resolution of EIT images can be improved in a very wide range of the rooting values as well as their optimums. This characteristic is very suitable for engineering applications.

(*3) Effectiveness.* The proposed method has been validated in four most used EIT imaging algorithms and can hold in most EIT algorithms.

(*4) Different Effects.* The SNR index is more suitable under noisy conditions, while the RS index works better in simulated or hardly free-noisy conditions when applying an EIT system. Moreover, to our knowledge, so far there is no quantity to measure the characteristics of investigated objects in various conditions, so these three quantities proposed in this paper are valuable for further setting a uniform criterion to compare various imaging processes and different algorithms.

There is a great room to improve the two proposed indexes. While promising, our immediate next studies will be as follows.

1. The use of the sensitive coefficients in the investigated field could generate vectors of more accurate characteristics to find more effective nonlinear tool instead of the rooting operation. The use of sensitive coefficients can provide better spatial resolutions, which has been demonstrated in the existing ET image algorithms.
2. The second study will be concerned with determining different weighting values for different components of all vectors. The existing study has shown that the measured data in different electrode pairs contain different noise-signal ratios and thus have different effects on the final EIT imaging results. Consequently, the use of different weight values to present these different effects is preferable to enhance the imaging spatial resolutions.
3. The third study will be on the use of other nonlinear transformations to measurable data besides the rooting operation. Various nonlinear transformation techniques have been developed for decades, and various research ways and achievements are rich and efficient.
